# Transitioning to da Vinci Xi for colorectal cancer surgery: a prospective cohort study of 102 cases from a UK centre with a structured robotic programme

**DOI:** 10.1007/s11701-025-02832-1

**Published:** 2025-09-29

**Authors:** Samuel Massias, Bhamini Vadhwana, Arian Arjomandi Rad, Lillian Reza, Joshua Franklyn, James Hollingshead, Najib Daulatzai, Vanash Patel

**Affiliations:** 1 Department of Surgery, West Hertfordshire Teaching Hospitals NHS Trust, Watford General Hospital, Vicarage Road, Watford, WD18 0HB UK; 2 Department of Surgery and Cancer, Imperial College London, St Mary’s Hospital, 10Th Floor QEQM Building, London, W2 1NY UK; 3 Oxford University Hospitals NHS Foundation Trust, Headley Way, Headington, Oxford, OX3 9DU UK; 4 Department of Colorectal Surgery, West Hertfordshire Teaching Hospitals NHS Trust, Watford General Hospital, Vicarage Road, Watford, WD18 0HB UK

**Keywords:** Colorectal surgery, Robotic assisted surgery, Training, Learning curves

## Abstract

This study evaluated short-term outcomes and learning curves following the introduction of the Intuitive© da Vinci Xi robotic platform for elective colorectal cancer resections at West Hertfordshire Teaching Hospital NHS Trust (WHTH). A smooth transition was enabled by prior experience with the CMR Surgical© Versius platform, with outcomes benchmarked against national data. A prospective cohort study included consecutive patients undergoing elective colorectal resections between April 2024 and March 2025. Data included demographics, diagnosis, operative details, complications, length of stay (LOS), and oncological outcomes. Results were compared with historical laparoscopic data from the National Bowel Cancer Audit (NBOCA, 2019–2022) and Model Health System (MHS, 2024). Learning curves for operative time were assessed using cumulative sum (CUSUM) analysis across three procedures: right hemicolectomy (RH), anterior resection (AR), and abdominoperineal resection (APR).

A total of 102 patients were included, with a median age of 69 years (IQR = 60–75), and 54.9% (*n* = 56) were male. All colonic resections (*n* = 72) achieved a lymph node yield ≥ 12, significantly higher than the 88.1% in NBOCA (*p* = 0.001). Among rectal resections (*n* = 30), 96.7% had negative margins versus 90.1% in NBOCA (*p* = 0.10). Conversion to open surgery was 3% (*n* = 3), the anastomotic leak rate was 1% (*n* = 1), and 4% (*n* = 4) required a return to theatre. MHS data showed that 13% of all colorectal patients at WHTH had a LOS ≥ 9 days, compared to 29% nationally (*p* = 0.0001), decreasing to 7.1% in the robotic cohort. CUSUM analysis showed stabilisation after ~ 12 right hemicolectomies and 20 low pelvic resections, with variability among surgeons. Surgeons with prior robotic experience achieved faster proficiency and generated time savings. The successful introduction of the da Vinci Xi platform at WHTH, supported by prior Versius experience, led to excellent oncological outcomes, shorter hospital stays, and low complication rates. These findings highlight the value of structured robotic implementation in advancing colorectal cancer care within the NHS.

## Introduction

Robotic-assisted surgery (RAS) was first introduced in the 1980s and was implemented in the United Kingdom (UK) during the 1990s. Since its initial adoption in cardiac surgery and urology, it has rapidly evolved into a multispecialty practice. The Commission on the Future of Surgery expects RAS to grow rapidly in popularity and present the most significant advancements in surgical care over the next 20 years, promoting the development of minimally invasive surgery and personalised treatments [1]. Higher surgical trainees in the UK are strongly motivated to integrate robotic-based training into the curriculum to reflect its growing utility in surgery [2]. Surgical robotics offers enhanced dexterity, greater precision, high definition 3-dimensional visualisation, and improved training potential. With the rapid progression of artificial intelligence (AI), the Royal College of Surgeons projects the integration of surgical robots capable of performing preprogrammed tasks to augment surgical practice [3].

In the UK, RAS has become an integral component of colorectal cancer care. Robotic systems are expanding into District General Hospitals (DGHs), matching established robotic services offered in teaching hospitals. Current literature demonstrates robotic-assisted bowel cancer resections are associated with reduced conversion rates and, in some studies, shorter operative times [4, 5]. Although early research generally reported no significant difference in oncological specimen quality between RAS and conventional laparoscopic surgery (CLS) [4, 6–8], more recent evidence suggests otherwise. A large randomised controlled trial (RCT) involving 1,171 patients with rectal cancer demonstrated a significant improvement in the completeness of resection and an increased number of harvested lymph nodes in the RAS group [9]. A recent meta-analysis involving 3193 patients reported a statistically significant reduction in severe complications and anastomotic leak rates among those undergoing RAS compared to those undergoing CLS [10]. Despite the high initial costs, RAS is cost-effective in the long term if specific implementation targets are met [11–13].

The da Vinci Surgical System by Intuitive^©^ was first introduced in the market following FDA approval in 2000  [14]. Da Vinci Xi, an advanced iteration, was released in 2014 and remains the most widely utilised multiport surgical robot globally, with over 6500 systems installed and more than 55,000 surgeons trained on the platform as of 2023  [14]. From the 2010s onwards, a new wave of competitors emerged, including CMR Surgical^©^ (Versius system), Medtronic^©^ (Hugo RAS system), and Johnson and Johnson^©^ (Ottava), reflecting growing international interest and competition in the field. This evolving sector will develop systems that are more ergonomic, compact, cost-effective, and increasingly integrated with AI, thereby transforming the future landscape of surgical practice.

This study aimed to [1] analyse the short-term outcomes of the first 102 elective colorectal cancer resections performed using da Vinci Xi, compared to previous CLS data, and [2] evaluate the learning curves associated with transitioning towards a new platform, following a 21-month program using the CMR Surgical^©^ Versius system.

## Methods

This research is registered with Research Registry and the identifying number is researchregistry11423 (https://www.researchregistry.com/browse-the-registry#home/).

The West Hertfordshire Teaching Hospitals NHS Trust (WHTH) is a high-volume surgical centre that commenced a phased implementation of the CMR Surgical^©^ Versius robotic system across several specialties, including colorectal, upper gastrointestinal, urology, and gynaecology, in 2022. Six colorectal consultants were trained on the Versius platform and began performing bowel cancer resections between July 2022 and April 2024, during which more than 100 procedures were undertaken [15]. In 2024, WHTH acquired the da Vinci Xi system, and four colorectal consultants were subsequently trained in its use, three of whom had previously operated using the Versius system. As of April 2024, colorectal resections have been performed at the Trust using the da Vinci Xi platform. Prior to 2022, all surgeons involved in this study were fully trained and experienced in conventional laparoscopic bowel cancer resections.

### Ethics approval

This project was not considered by the Health Research Authority (HRA). Therefore, HRA research ethics approval was not required for this study. The service evaluation was reviewed and approved by the information governance team and clinical lead for surgery at the WHTH prior to its commencement.

### Patient recruitment and data collection

A total of 102 patients who underwent elective bowel cancer resection using the da Vinci Xi surgical system, performed by four colorectal consultants, were recruited from April 2024 to May 2025. Clinical data collected from electronic patient records included patient demographics, diagnosis and staging, preoperative comorbidities, operative details (type of procedure and duration), length of hospital stay, estimated blood loss, analgesic requirements, histological findings, and perioperative and postoperative complications. The data were compared with existing CLS outcomes published by the National Bowel Cancer Audit (NBOCA) from 2019 to 2022 [16]. Our institution is a teaching hospital. All consultants completed the Intuitive training pathway before undertaking independent cases, with initial proctored cases (included in this series). Once beyond their learning curve, consultants began training senior trainees through our structured robotic programme, with trainee involvement always under direct supervision [17].

### Statistical analysis

Data were processed and analysed in IBM SPSS Statistics (version 29), with results cross-checked in R (v4.4.0) to ensure reproducibility. Two complementary analytic streams were followed. First, traditional hypothesis-testing compared outcomes between the robotic and historical laparoscopic cohorts. Continuous variables were inspected for normality with Shapiro–Wilk tests and Q–Q plots; those departing from normality are reported as median (inter-quartile range) and compared with the Mann–Whitney U test, whereas categorical variables—including sex, conversion, anastomotic leak, and Clavien–Dindo grade ≥ III complications—were examined using Pearson’s *χ*^2^ test or Fisher’s exact test when expected counts fell below 5. A two-sided *p* < 0.05 denoted statistical significance, and because this was an exploratory service-evaluation, no adjustment for multiplicity was applied; 95% confidence intervals (CI) accompany all estimates to convey precision.

Second, the surgical learning curve was modelled with a cumulative‑sum (CUSUM) approach to track operative efficiency over time. Individual OPCS‑4 procedure codes were collapsed into three families—right or extended right hemicolectomy, (low) anterior resection, and abdominoperineal resection—to maximise statistical power. Learning curves for other operations, such as left hemicolectomy and sigmoid colectomy, were not analysed owing to low case numbers. For each family, the overall mean operative time served as the reference target (T). For every case *i*, the deviation *d*_*i*_ was calculated as operative time minus T, and the running CUSUM at case *n* equalled the sum of all deviations up to n. Upward slopes therefore indicated cumulative excess time characteristic of early learning, whereas downward slopes signalled time savings consistent with growing proficiency. Two performance landmarks were derived: the peak (turning‑point) case, defined as the first global maximum after which the curve began to descend, and the baseline‑return case, when the CUSUM crossed ≤ 0, marking time‑neutral proficiency. Sensitivity analyses included Winsorising operative times at the 95th percentile and constructing risk‑adjusted CUSUM curves that removed variance attributable to age, BMI, and ASA grade.

All graphs were produced in ggplot2, with 95% CIs smoothed by LOESS where appropriate. Source code and anonymised data are available from the corresponding author on reasonable request.

## Results

### Patient clinical data

A total of 102 patients underwent elective bowel cancer resection by using the da Vinci Xi surgical system. Of these, 54.9% (*n* = 56) were male, with a median age of 69 years (interquartile range [IQR] = 60–75). Reported ethnic classification showed 74.5% (*n* = 76) of patients were ‘White British,’ 8.8% (*n* = 9) ‘White Other,’ 2% (*n* = 2) ‘Asian or Asian British,’ and 1% (*n* = 1) ‘Black or Black British.’ The mean BMI was 27.3 (standard deviation [SD] = 4.9). The mean ASA grade was 2.3 (SD = 0.4), the Charlson Comorbidity Index (CCI) was 5.2 (SD = 1.7), and the Colorectal Physiological and Operative Severity Score for the Enumeration of Mortality and Morbidity (CR-POSSUM) was 16.5 (SD = 2.3). A mean CCI of 5.2 corresponds to an estimated 10-year survival probability of 22%, whilst the mean CR-POSSUM equates to a predicted mortality risk of 2.4% [18].

Patient tumour locations included rectal adenocarcinoma (confirmed by Magnetic Resonance Imaging and histology) in 29.1% (*n* = 30), sigmoid adenocarcinoma in 22.1% (*n* = 22), ascending colon carcinoma in 14.6% (*n* = 15), and caecal carcinoma in 13.6% (*n* = 14). Synchronous tumours identified during preoperative investigations were reported in one patient (1%). Neoadjuvant chemoradiotherapy was administered to 40% (*n* = 12) of patients with rectal adenocarcinoma and 4.5% (*n* = 1) with sigmoid adenocarcinoma. The highest volume surgeon performed 34 cases, whilst the lowest volume surgeon undertook 20 cases. The most common operation performed was a right hemicolectomy (39.2%, n = 40), followed by an anterior resection (29.4%, *n* = 30), abdominoperineal resection (14.7%, *n* = 15), and left hemicolectomy (9.8%, *n* = 10). Patient clinical data and operative details are shown in Table 1.

**Table 1:** Patient characteristics (*n* = 102) undergoing elective RAS colorectal cancer resections using da Vinci Xi

	Number, *n* (%)
Age	69 (IQR 15)
Gender (Male)	56 (54.9%)
Ethnicity	
White British	76 (74.5%)
White other	9 (8.8%)
Asian or Asian British	2 (2%)
Black or Black British	1 (1%)
Other / Not stated	12 (11.8%)
ASA	
Grade 2	75 (73.5%)
Grade 3	27 (26.5%)
Body mass index	
x < 25	31 (30.7%)
25 ≤ x < 30	44 (43.6%)
30 ≤ x < 35	20 (19.8%)
35 ≤ x < 40	3 (3%)
x > 40	3 (3%)
CR-POSSUM	
13 ≤ x ≤ 15	35 (34.3%)
16 ≤ x ≤ 18	51 (50%)
19 ≤ x ≤ 21	11 (10.8%)
22 +	5 (4.9%)
Cancer diagnosis	
Rectal adenocarcinoma	30 (29.1%)
Sigmoid adenocarcinoma	22 (21.4%)
Ascending colon adenocarcinoma	15 (14.6%)
Caecal adenocarcinoma	14 (13.6%)
Descending colon adenocarcinoma	7 (6.8%)
Hepatic flexure adenocarcinoma	6 (5.8%)
Transverse colon adenocarcinoma	6 (5.8%)
Splenic flexure adenocarcinoma	2 (1.9%)
Terminal ileal neuroendocrine tumour	1 (1%)
Neoadjuvant chemoradiotherapy*	
Rectal cancer (*n* = 30)	12 (40%)
Colon cancer (*n* = 72)	1 (1.4%)
Procedure name	
Right hemicolectomy	40 (39.2%)
Abdominoperineal resection	16 (15.7%)
High anterior resection	10 (9.8%)
Low anterior resection	14 (13.7%)
Left hemicolectomy	10 (9.8%)
Sigmoid colectomy	8 (7.8%)
Subtotal colectomy	3 (2.9%)
Extended right hemicolectomy	1 (0.9%)

^*^Both short-course and long-course radiotherapy were used depending on the patient’s circumstances

### Patient outcomes

Of the 102 procedures performed using the da Vinci Xi system, 2.9% (*n* = 3) were converted to open surgeries. The three documented reasons for conversion included mesenteric bleeding in a patient with T4N2M0 caecal adenocarcinoma undergoing a right hemicolectomy with concurrent Crohn’s disease (the only patient with inflammatory bowel disease in the cohort), brisk presacral haemorrhage, and dense intra-abdominal adhesions. The mean BMI of patients requiring conversion was 31.1 (standard deviation [SD] = 3.5). A total of 7 (6.9%) patients experienced postoperative complications classified as Clavien-Dindo grade 3a or higher. The anastomotic leak rate was 1% (*n* = 1) needing a return to theatre and subsequently accounting for the single mortality in this cohort. Three other returns to theatre included persistently high drain output, stoma necrosis, and port-site hernia. One patient underwent radiologically guided drainage of an intra-abdominal hematoma under local anaesthesia. Two patients sustained intraoperative iatrogenic visceral injuries, one to the small bowel and one to the bladder. Both were identified and repaired during surgery, and no postoperative complications were documented. A total of 75.5% of patients (*n* = 77) had no reported complications. The mean postoperative follow-up duration for all patients was 97.8 days postoperatively (SD = 50.5).

The mean in-hospital length of stay (LOS) was 4.2 days (SD = 2.4), with a risk-adjusted LOS of more than 5 days of 23.4% (95% confidence interval (CI) 15.5–32.9%). The percentage of patients with a risk-adjusted LOS ≥ 9 days or higher was 7.1% (95% CI 2.3–13%). The proportion of patients with a risk-adjusted length of stay greater than five days was significantly lower in the RAS cohort (23%) compared to the CLS cohort reported by NBOCA (39%) (*p* = 0.001). The Model Health System (MHS) reports that the proportion of all patients who underwent an elective bowel resection for colorectal cancer at WHTH between January 2024 and December 2024, staying in hospital 9 days or longer was 12.6%, compared to the national average of 29% (*p* = 0.0001). This is an improvement from January 2023 to December 2023, when the proportion at WHTH was 23.3% [19]. The percentage of patients in our RAS cohort with a risk-adjusted LOS of 9 days or higher was even lower at 7.1% (95% CI 2.3–13%). The mean lymph node yield across all bowel specimens was 26.4 (SD = 8.9). The mean lymph node yield was 26.1 (SD 8.1) for right hemicolectomies, 19.0 (SD 5.1) for left hemicolectomies, 25.4 (SD 9.1) for anterior resections, and 22.1 (SD 7.6) for abdominoperineal resections. All the patients included in the study had a lymph node yield of ≥ 12. Negative resection margins were achieved in 98% of all specimens (*n* = 100) and in 96.7% of rectal cancer cases (*n* = 29). The first positive margin occurred in a patient with T4N2M0 caecal adenocarcinoma, with 16 of 22 lymph nodes positive. The second was in a patient with T3N2M0 rectal adenocarcinoma, who had received no neoadjuvant therapy and had 11 of 37 nodes positive. The negative resection margin rate for rectal cancers using the da Vinci Xi system showed a 6% improvement compared to the Trust’s CLS figures reported by NBOCA, although this difference was not statistically significant (*p* = 0.10). However, the rate was 14% higher than the corresponding national figure of 83% for the same period (2019–2022), which was statistically significant (*p* = 0.02). Among all patients who underwent surgery, the mean reduction in haemoglobin was 12.3 g/L (SD 10.1) between pre- and immediate post-operative blood tests, and 4.9% (*n* = 5) required a blood transfusion during admission. A comparison of key performance indicators between the da Vinci Xi robotic cohort and the historical laparoscopic outcomes reported by the NBOCA is presented in Table 2.

**Table 2:** Outcomes from the key quality performance indicators of 100 patients undergoing RAS with da Vinci Xi compared to conventional laparoscopic surgery (NBOCA, *n* = 442)

	CLS 2019–2022 (Trust)	LS 2019–2022 (National)	da Vinci Xi RAS 2024–2025
	Proportion (%)	Proportion (%	Proportion (%)
	Total *n* = 442	Total *n* = 53,984	Total *n* = 102
	Rectal *n* = 130	Rectal *n* = 11,869	Rectal *n* = 30
Lymph node yield ≥ 12 (colon)	88%	85%	100% (*p* = 0.001)
Negative margin rate (rectal)	91%	83%	97% (*p* = 0.10)
Risk adjusted length of stay > 5 days (all cases)	39%	56%	23% (*p* = 0.001)

Stated *P*-values compare data between CLS 2019–2022 (Trust) and da Vinci Xi RAS 2024–2025 outcomes

### CUSUM learning curve analysis

#### Individual CUSUM analysis

Out of the four surgeons involved in this study, *Surgeon 1*, *Surgeon 2*, and *Surgeon 3* had prior experience with Versius by CMR Surgical. *Surgeon 1* (25 patients) showed the greatest improvement in all families. For right hemicolectomy (RH) and anterior resection (AR), the CUSUM peaked in the first two cases and crossed below zero in the 2nd–3rd case, after which cumulative savings exceeded 5 h per family (mean operative times 14–25% shorter than the institutional reference). *Surgeon 2* quickly attained proficiency in RH (case 2) and AR (case 3) but recorded only two abdominoperineal resection (APR) cases, remaining 64 min above the target. A small upward drift after returning to the baseline in AR left a modest positive net CUSUM (+ 25 min). *Surgeon 3* cleared the deficit in APR in the 2nd case but did not achieve time-neutral performance in AR or RH. The AR curve continued to rise across all three cases (+ 248 min), and the RH curve peaked in Case 12 (+ 162 min) without crossing zero. *Surgeon 4* logged no cases of APR. Their AR and RH curves both peaked late (cases 3 and 7, respectively) and remained above baseline until the end of follow-up, indicating an ongoing learning phase (net excess + 38 min AR, + 279 min RH). With regard to the speed of adoption, two surgeons (1 and 2) reached proficiency in RH in two cases, whilst only *Surgeon 1* achieved durable efficiency gains in all three procedure families. Time to proficiency ranged from 2 to > 7 cases for RH, and from 3 to > 3 cases (not achieved) for AR. APR estimates are imprecise because of their low volume. We found that across the study, surgeons 1 and 2 generated net time savings (negative final CUSUMs), whereas surgeons 3 and 4 accumulated excess operative time in one or more families.

#### Cumulative CUSUM analysis

**Fig. 1:**
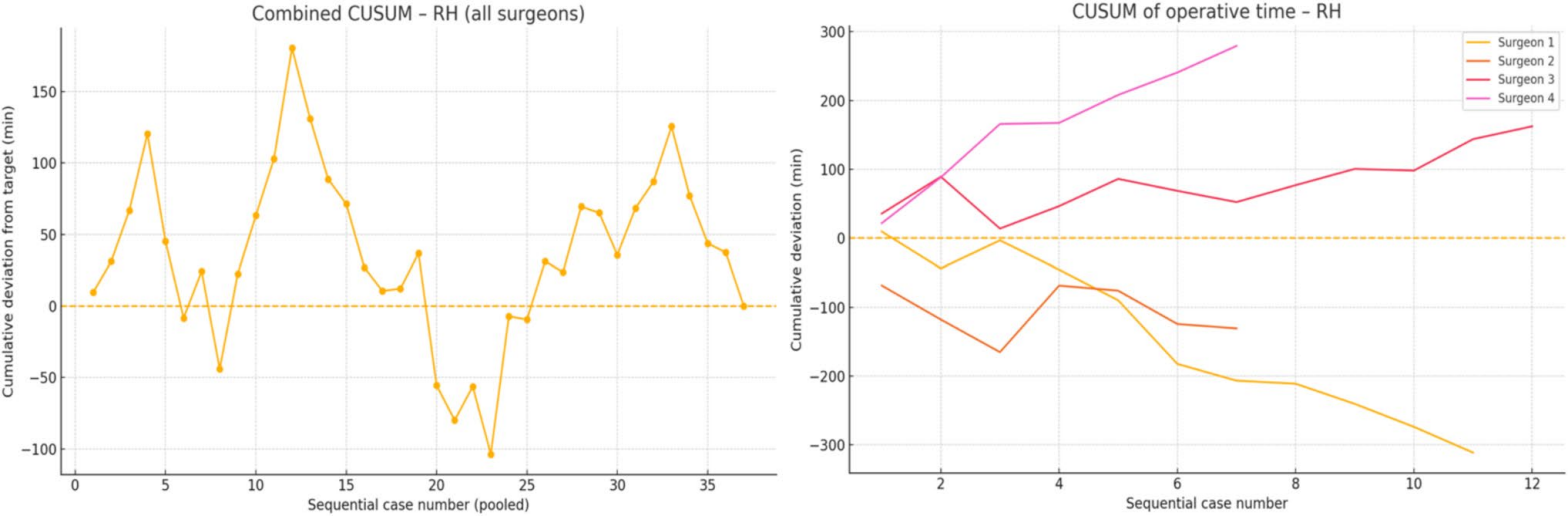
CUSUM analysis of operative times for right hemicolectomies: all surgeons combined (left) and individual surgeon data (right)

**Fig. 2:**
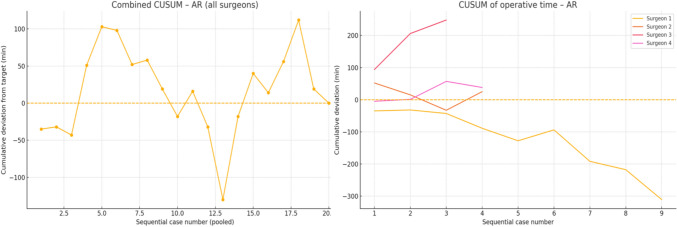
CUSUM analysis of operative times for anterior resections: all surgeons combined (left) and individual surgeon data (right)

**Fig. 3:**
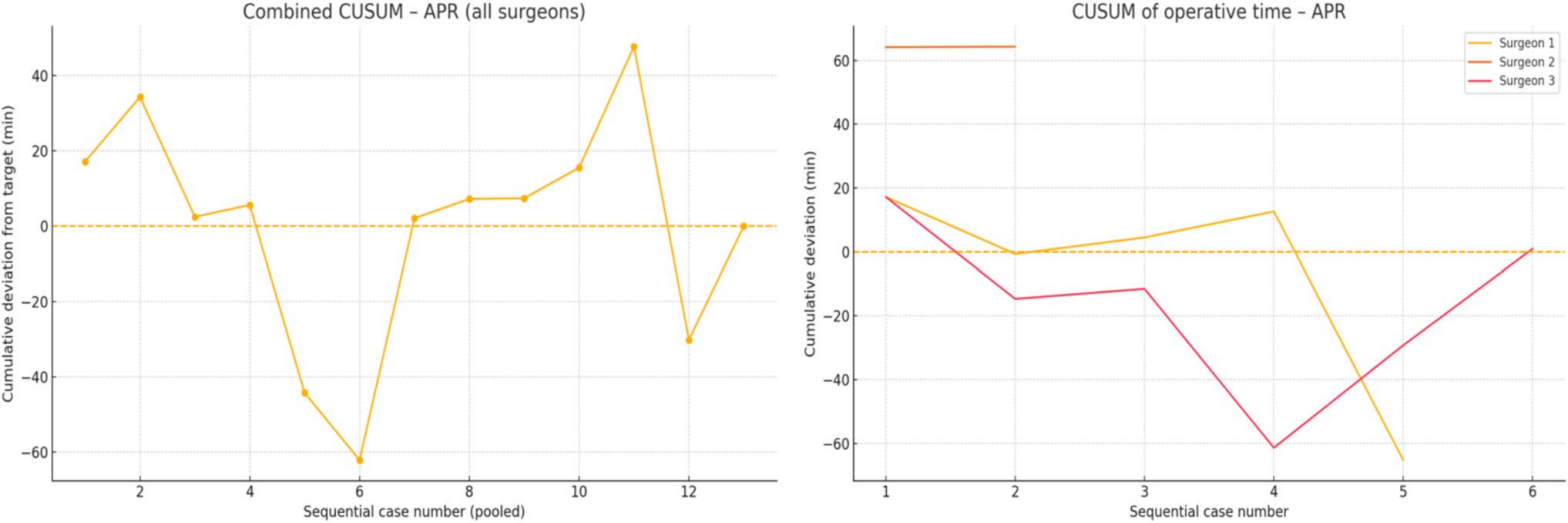
CUSUM analysis of operative times for abdominoperineal resections: all surgeons combined (left) and individual surgeon data (right)

For RH, there was a steep early climb, reaching the largest excess (+ 180 min) in Case 12. An extended downturn between cases 13 and 20 eliminated the deficit, and the curve oscillated around zero thereafter. For AR, CUSUM climbed steadily and peaked late (Case 18). Two subsequent cases cleared the accumulated 100 min surplus, bringing the curve back to the baseline in Case 20. For APR, there was a small series. The CUSUM rose gradually to a modest peak in Case 11, then fell below the baseline in Case 12, indicating that the unit regained time-neutral performance within one additional case. Overall, the service required approximately 12 cases to stabilise right-sided resections and 20 cases for low-pelvic resections. Once the baseline was re-established, the net operative time for each family member was neutral at the end of the study window. The cumulative and individual CUSUM analyses are displayed in Fig. 1 for RH, Fig. 2 for AR, and Fig. 3 for APR.

## Discussion

The primary findings of this study demonstrate that the implementation of the da Vinci Xi system at our trust has been safe and effective, resulting in clinical outcomes that are non-inferior to those achieved with conventional laparoscopic techniques and superior in certain parameters such as lymph node yield and LOS. These results remain consistent following the initial 21-month period of use of the Versius system by CMR Surgical^©^. According to the MHS, 76.9% of elective bowel cancer resections at WHTH were performed robotically between January and December 2024, markedly higher than the national average of 26.7% for the same period  [19]. WHTH has seen a significant increase in RAS utility, which accounted for only 20.8% of elective bowel cancer resections between January and December 2022  [19]. These findings indicate that the rapid implementation of RAS within a DGH setting can be achieved safely with strategic planning and multidisciplinary collaboration.

A significant improvement in lymph node yield was observed with the use of the da Vinci Xi system compared with CLS, as reported by NBOCA, with the key performance indicator of 12 or more lymph nodes achieved in 100% of the resections performed. This represents a further improvement from the 95.3% rate achieved with the Versius system between 2022 and 2023  [15], likely reflective of improved techniques with the robotic platform. Alternatively, it could be argued that the learning curve had been fully or partially attained with the previous platform, as current evidence demonstrates proficiency in lymph node yield in approximately 50 cases [20]. A study of 2398 patients reported that RAS was associated with a higher lymph node yield than both conventional laparoscopic and open colorectal resections [21], with a clear translation to improved disease-free and overall survival [22, 23]. Although our data showed no substantial improvement in achieving negative margin rates, the REAL multicenter RCT involving 1090 patients with low to mid-rectal cancer demonstrated that those undergoing robotic surgery were significantly less likely to have a positive circumferential resection margin than those treated laparoscopically [9].

Our study demonstrated a reduction in the LOS among patients who underwent resections using the da Vinci Xi system compared to those who had CLS, translating to an estimated cost saving of £407 per inpatient bed day [24]. A potential confounding variable that may have contributed to the improvement in LOS was the parallel implementation of the Virtual Hospital (VH) at WHTH in November 2023, whereby suitable patients were enrolled in an early discharge pathway with remote monitoring at home by specialist nursing staff. In addition, the introduction of robotic intracorporeal anastomosis may also have influenced outcomes and represents another potential confounder [25]. Twenty-eight (27.5%) patients in this cohort were discharged via the VH pathway. Our findings suggest a significant difference in the length of hospital stay between the robotic and laparoscopic surgery groups, which contrasts with current literature findings [26, 27]. We propose that RAS, alongside a VH setting, can facilitate early discharge in patients achieving early optimisation with pain relief and mobility with minimally invasive surgery.

A meta-analysis involving 53,329 patients undergoing laparoscopic colorectal cancer resections reported a median conversion rate of 14.3%, with rates of 12.8% for colonic and 10% for rectal resections [28]. Our 3% conversion rate is in keeping with the literature. Six patients in the cohort had a BMI greater than 35, none of whom required conversion to open surgery or return to theatre. Our experience supports the idea that the da Vinci Xi system can be employed effectively in technically challenging cases. There is a cost saving to NHS, where conversion to open surgery is estimated at £2,221 [29] and anastomotic leak is estimated at £7,137 [30]. Our anastomotic leak rate using the da Vinci Xi system was low, at 1% (*n* = 1). Current literature also reports low leak rates with conventional laparoscopic surgery, with a systematic review of 23,568 patients and a rate of 2.8% for laparoscopic resections [31]. Our observed mortality rate of 1% at a mean follow-up of 97.8 days was lower than the predicted in-hospital or 30-day mortality of 2.4%, as estimated by the CR-POSSUM score.

Analysis of sequential operative times using the da Vinci Xi system demonstrated minimal or no significant improvement, likely reflecting the prior robotic experience of three of the four participating surgeons with the Versius system. CUSUM analysis revealed considerable inter-surgeon variation in the rate at which operative proficiency was attained across different procedures. Notably, Surgeons 1 and 2, both of whom achieved net time savings over the course of the study, had previous experience operating with the Versius system by CMR Surgical^©^ prior to transitioning to the da Vinci Xi. This observation aligns with the existing literature suggesting that console-operating skills may be transferable across different robotic platforms [32]. At the departmental level, our findings indicate that operative times progressively stabilise with increasing case volume. Although differences in instrumentation, port placement, docking, laparoscopic assistance, and cost exist between robotic platforms, the present study was not designed as a head-to-head comparison between platforms. A forthcoming analysis will provide a comprehensive evaluation of inter-platform differences and the strategic rationale behind our institutional transition.

Whilst this study provides valuable early insights into the adoption of RAS using the da Vinci Xi system in a DGH setting, certain limitations should be acknowledged. The study included a relatively modest sample size of 102 patients with a non-contemporaneous laparoscopic comparator group. Furthermore, although a significant reduction in the length of hospital stay was observed, this outcome may have been partly influenced by the concurrent service development of the VH discharge pathway. Beyond technical outcomes, several lessons emerged from our adoption process. Critical enablers included structured training pathways for surgeons, tailored education for operating room staff, and agreed protocols for case selection. Engagement with hospital leadership was essential to address concerns about initial costs and operating time. Monitoring learning curves proved valuable, and future programmes should consider targeted interventions for surgeons with slower progress.

## Conclusions

This study demonstrated that implementing RAS using the da Vinci Xi within a DGH setting is feasible and can be safely achieved with positive patient outcomes. The observed low complication and conversion rates, along with improved oncological quality indicators, affirmed the clinical efficacy of the platform. Despite the initial high capital expenditure, a reduction in the length of hospital stay, complications, and oncological-related costs indicates substantial long-term economic benefits. These findings support a growing body of evidence that, with strategic planning, multidisciplinary collaboration, and investment in comprehensive training, the broader incorporation of robotic surgical platforms across diverse healthcare settings is both practical and sustainable. Consequently, RAS represents a viable advancement for future colorectal cancer care in the UK.

## Data Availability

The datasets generated and/or analysed during the current study are available from the corresponding author on reasonable request.
